# Transcriptional profiling of paediatric ependymomas identifies prognostically significant groups

**DOI:** 10.1002/cjp2.236

**Published:** 2021-07-27

**Authors:** Maria Łastowska, Ewa Matyja, Anna Sobocińska, Bartosz Wojtaś, Magdalena Niemira, Anna Szałkowska, Adam Krętowski, Agnieszka Karkucińska‐Więckowska, Magdalena Kaleta, Maria Ejmont, Magdalena Tarasińska, Marta Perek‐Polnik, Bożenna Dembowska‐Bagińska, Maciej Pronicki, Wiesława Grajkowska, Joanna Trubicka

**Affiliations:** ^1^ Department of Pathomorphology The Children's Memorial Health Institute Warsaw Poland; ^2^ Department of Experimental and Clinical Neuropathology Mossakowski Medical Research Institute, Polish Academy of Sciences Warsaw Poland; ^3^ Neurobiology Center Nencki Institute of Experimental Biology Warsaw Poland; ^4^ Clinical Research Centre Medical University of Białystok Białystok Poland; ^5^ Clinic of Oncology The Children's Memorial Health Institute Warsaw Poland

**Keywords:** ependymoma, molecular groups, NanoString, prognosis, transcriptional profiling

## Abstract

The majority of supratentorial ependymomas in children contain oncogenic fusions, such as *ZFTA–RELA* or *YAP1‐MAMLD1*. In contrast, posterior fossa (PF) ependymomas lack recurrent somatic mutations and are classified based on gene expression or methylation profiling into group A (PFA) and group B (PFB). We have applied a novel method, NanoString nCounter Technology, to identify four molecular groups among 16 supratentorial and 50 PF paediatric ependymomas, using 4–5 group‐specific signature genes. Clustering analysis of 16 supratentorial ependymomas revealed 9 tumours with a *RELA* fusion‐positive signature (RELA+), 1 tumour with a *YAP1* fusion‐positive signature (YAP1+), and 6 not‐classified tumours. Additionally, we identified one RELA+ tumour among historically diagnosed CNS primitive neuroectodermal tumour samples. Overall, 9 of 10 tumours with the RELA+ signature possessed the *ZFTA‐RELA* fusion as detected by next‐generation sequencing (*p* = 0.005). Similarly, the only tumour with a YAP1+ signature exhibited the *YAP1‐MAMLD1* fusion. Among the remaining unclassified ependymomas, which did not exhibit the *ZFTA‐RELA* fusion, the *ZFTA‐MAML2* fusion was detected in one case. Notably, among nine ependymoma patients with the RELA+ signature, eight survived at least 5 years after diagnosis. Clustering analysis of PF tumours revealed 42 samples with PFA signatures and 7 samples with PFB signatures. Clinical characteristics of patients with PFA and PFB ependymomas corroborated the previous findings. In conclusion, we confirm here that the NanoString method is a useful single tool for the diagnosis of all four main molecular groups of ependymoma. The differences in reported survival rates warrant further clinical investigation of patients with the *ZFTA‐RELA* fusion.

## Introduction

The integrated use of whole‐genome analysis technologies has revealed the site‐specific molecular heterogeneity of ependymomas. The majority of supratentorial tumours contain oncogenic fusions between the genes *C11orf95* and *RELA* (*C11orf95–RELA*) or *YAP1* (*YAP1‐MAMLD1*) [1, 2]. Notably, the gene *C11orf95* is now called *ZFTA* (Zinc Finger Translocation Associated), and this name is used hereafter. In contrast, posterior fossa (PF) tumours lack recurrent somatic mutations and are most likely driven by epigenetic events. These tumours are classified according to gene expression or methylation profiling into two broad groups: group A (PFA) and group B (PFB) [2, 3, 4]. Tumour molecular characterisation is clinically relevant, as tumours with *ZFTA–RELA* translocation and PFA ependymomas are reportedly associated with poor prognosis [1, 3]. The diagnostic category of *RELA* fusion‐positive ependymoma was therefore introduced in the most recent 4th edition of the *WHO Classification of Tumours of the Central Nervous System* [5].

Clinically relevant molecular subtypes of ependymoma have been identified using a variety of methods, including gene expression microarrays [3, 6], methylation profiling [3, 4, 7], reverse transcription PCR for *ZFTA‐RELA* fusion mRNA [1], and immunohistochemistry [3]. We have applied a novel and potentially diagnostic approach for the identification of four molecular groups of ependymoma, based on transcription profiling of marker genes, using NanoString nCounter Technology (NanoString Technologies, Seattle, WA, USA). This method enables the analysis of degraded RNA, and is thus compatible with formalin‐fixed paraffin‐embedded (FFPE) tumour samples. It has previously been successfully tested for the identification of molecular subtypes in medulloblastoma, and the diagnosis of rare paediatric brain tumours [8, 9].

In the present study, we analysed 16 supratentorial and 50 PF tumours, and identified four molecular types of ependymoma. Analysis of the clinical characteristics of these patients confirmed previous findings for PFA and PFB tumours. However, the majority of patients in our series with *ZFTA‐RELA* fusion exhibited a good prognosis, indicating a need for further clinical investigation of this molecular group.

## Materials and methods

### Patients and tumour material

This analysis included paediatric patients diagnosed with ependymomas, CNS embryonal tumours ‘not otherwise specified’ (NOS), and CNS primitive neuroectodermal tumours (CNS‐PNETs) category, as diagnosed before the WHO 2016 classification was introduced, at The Children's Memorial Health Institute in Warsaw, Poland, between 1996 and 2019. Analysis was performed using archived FFPE tumour material obtained at diagnosis. Two experienced neuropathologists and one paediatric pathologist performed histopathological evaluation of haematoxylin and eosin‐stained slides to verify the original diagnosis of ependymoma and to determine tumour tissue content. Whole preparations were scanned using a Hamamatsu NanoZoomer 2.0 RS scanner (Hamamatsu Photonics, Hamamatsu, Japan) at an original magnification of ×40. Only samples that contained >70% of tumour cells were investigated.

After verification, our subsequent analysis included 16 supratentorial ependymomas, 50 PF ependymomas, and 7 CNS‐PNETs or CNS embryonal tumours NOS. The supratentorial ependymomas included two reference tumours – one that was *ZFTA‐RELA* fusion‐positive and another that was *ZFTA–RELA* fusion‐negative, as detected by next‐generation sequencing (NGS) prior to NanoString analysis.

Tumours were retrospectively analysed and tissues were retrieved from the archives of the Pathomorphology Department of the Children's Memorial Health Institute's, Warsaw, Poland, under the agreement from The Bioethics Committee at the Children's Memorial Health Institute.

### Identification of group‐specific marker genes by microarray *in silico* analysis

We re‐analysed a total of 345 CEL files deposited in the Gene Expression Omnibus (GEO) database. These included only paediatric cases (patients <18 years old) that were investigated using the Affymetrix Human Genome U133 Plus 2.0 platform (Affymetrix, Santa Clara, CA, USA).

We compared the 36 *RELA* fusion‐positive (RELA+) and 5 *YAP1* fusion‐positive (YAP1+) ependymomas from the GSE64415 set [2] to other supratentorial tumours. These included 12 atypical teratoid rhabdoid tumours (ATRTs) from the GSE73038 [10] and GSE70678 [11] sets, as well as the following tumours from the GSE73038 set: 29 high‐grade gliomas (HGGs), 6 embryonal tumours with multilayered rosettes (ETMRs), 9 CNS neuroblastomas with *FOXR2* activation (CNS NB‐FOXR2), 5 CNS Ewing sarcoma family tumours with *CIC* alteration (CNS EFT‐CIC), 6 CNS high‐grade neuroepithelial tumours with *MN1* alteration (CNS HGNET‐MN1), and 8 CNS high‐grade neuroepithelial tumours with *BCOR* alteration (CNS HGNET‐BCOR).

We compared 62 PFA and 7 PFB ependymomas from the GSE64415 set with 99 medulloblastomas from the GSE73038 and GSE10327 [12] sets, 21 PF ATRTs from the GSE73038 and GSE70678 sets, and 40 other infrequently found tumours in the PF from the GSE73038 set – namely, K27 HGGs, ETMRs, CNS HGNET‐BCORs, and CNS HGNET‐MN1 tumours.

CEL files were uploaded to the R environment and subsequently normalised with the MAS5 method using the ‘affy’ library. Next, quantile normalisation was performed separately on supratentorial and infratentorial tumours. Data were log2 transformed, and affy probes with the lowest variance (variance < 0.25) were filtered out. In a supervised approach, affy probes were selected using Student's *t*‐test. A set of samples with ependymoma molecular subtype was compared 100 times to an equal‐sized set drawn from patient samples with different diagnoses. The mean *P* value and mean fold‐change from 100 *t*‐tests were used as measures of a good marker candidate. We performed random selection of samples and repeated the *t*‐test 100 times to assure the robustness of marker selection.

### Detection of molecular groups at the RNA level

Total RNA was extracted from FFPE tumour samples using RNeasy kits (Qiagen, Hilden, Germany). For the identification of four molecular subgroups (RELA+, YAP1+, PFA, and PFB), we applied NanoString nCounter system analysis (NanoString Technologies) in a series of 66 ependymomas and 7 PNET or CNS embryonal tumours NOS. For group assignment, we applied a custom NanoString CodeSet that included marker genes and three housekeeping genes (*ACTB*, *GAPDH*, and *TBP*).

Probes were designed to target the regions of the marker genes (the sequences are presented in supplementary material, Table S1). Hybridisation of these probes to the tumour RNA samples was performed in the Clinical Research Centre, Medical University of Białystok, Poland, following NanoString Technologies procedures for hybridisation, detection, and scanning. The raw counts for each gene were subjected to technical and biological normalisation using nSolver 4.0 software (NanoString Technologies). Clustering of the samples was performed using Euclidean distance metrics and average settings.

### Detection of *RELA* and *YAP1* fusions via targeted NGS

*RELA* and *YAP1* gene fusions were detected using targeted cancer panel sequencing – the Archer FusionPlex Solid Tumour Panel (Archer Dx, Boulder, CO, USA) and/or the Ampliseq Childhood Cancer Panel for the Illumina assay (Illumina, San Diego, CA, USA). Prior to library preparation, total RNA was extracted from FFPE tumour samples using the RNeasy Mini Kit (Qiagen), and then quantified with the QuantiFluor RNA system (Promega, Madison, WI, USA). Post‐extraction analyses included additional quality control metrics to establish cut‐offs for RNA concentration and quality. RNA purity was evaluated using a Nanodrop spectrophotometer (Thermo Fisher Scientific, Waltham, MA, USA) for optical density (OD) 260/280 ratios. The percentage of RNA fragments >200 nucleotides (DV200) was calculated using an Agilent 2100 Bioanalyzer. Samples with an OD 260/280 ratio of >1.6 and with DV200 scores of >30% were considered acceptable for downstream processing. We used approximately 20 ng RNA for library construction, according to the manufacturer's protocols. Each library was assessed qualitatively using an Agilent 2100 Bioanalyzer (Agilent Technologies, Santa Clara, CA, USA), and quantified using the QuantiFluor RNA system. Then, the obtained library was amplified using universal primers targeting the paired‐end adapters. Clusters were generated and sequenced on the MiniSeq instrument (Illumina, San Diego, CA, USA) using the MiniSeq High‐Output Kit (2 × 150 cycles). FASTQ files with base call and quality information of a minimum of 4.5 million paired‐end sequence reads were processed using Archer Analysis Software (Suite_Analysis_v6.2.7) and/or the BaseSpace RNA Amplicon workflow (Illumina) to determine the presence of *RELA* and *YAP1* fusion genes.

### Statistical analysis

Statistical analyses were performed using the *t*‐test and Fisher's exact test. Overall survival (OS) and progression‐free survival (PFS) were calculated using Kaplan–Meier estimates, and group comparisons were made using the log‐rank test. Analyses were performed using SPSS software (version 26; SPSS Inc., Chicago, IL, USA). All patients had a follow‐up period of at least 2 years.

## Results

### Identification of group‐specific marker genes

Group‐specific candidate marker genes were selected based on both fold‐change and statistical significance. Table 1 presents the top up‐regulated probes/genes for each category compared to all other types of tumour. This list includes probes/genes with at least a fivefold increase in expression level and statistical significance within the top 20 positions. For further analysis, in addition to the marker genes, 4–6 candidate genes were selected among the most significantly up‐regulated probes for each group, excluding unknown and microRNA coding genes. We also investigated four genes for identification of the molecular subgroups PFA1 (*SKAP2* and *WIF1*) and PFA2 (*EN2* and *CNPY1*) [7].

**Table 1:** Marker genes selected for identification of molecular groups in ependymoma.

Group	Probe	Gene	Top fold	Significance top position
RELA+	201783_s_at	*RELA*	Group marker	
	206844_at	*FBP2*	716.4	13
	223967_at	*ANGPTL6*	132.0	1
	221400_at	*MYO3A*	49.5	10
	241382_at	*PCP4L1*	42.7	12
	244364_at	*MYO3A*	40.7	8
	219517_at	*ELL3*	26.1	7
	228994_at	*CCDC24*	20.9	9
	219518_s_at	*ELL3*	20.4	4
YAP1+	231729_s_at	*CAPS*	215.1	10
	214652_at	*DRD1*	113.6	4
	209540_at	*IGF1*	62.6	18
	231728_at	*CAPS*	43.8	3
	209542_x_at	*IGF1*	37.0	13
	227848_at	*PEBP4*	35.6	12
	211577_s_at	*IGF1*	27.0	15
	1554097_a_at	*MIR31HG*	17.6	1
	236940_at	*NA*	16.7	5
	213085_s_at	*WWC1*	13.8	8
	1554044_a_at	*MRAP*	12.5	2
PFA	205116_at	*LAMA2*	Group marker	
	207695_s_at	*IGSF1*	60.9	2
	227848_at	*PEBP4*	54.1	17
	224339_s_at	*ANGPTL1*	50.7	16
	237058_x_at	*SLC6A13*	38.9	5
	239183_at	*ANGPTL1*	36.7	20
	231063_at	*NA*	35.7	13
	1555396_s_at	*CXorf67*	26.8	1
	244084_at	*AIFM3*	20.6	7
	203571_s_at	*ADIRF*	19.3	12
	1557286_at	*NA*	18.7	11
	226420_at	*MECOM*	16.5	19
	221884_at	*MECOM*	16.1	8
	205208_at	*ALDH1L1*	10.0	3
PFB	203413_at	*NELL2*	Group marker	
	232005_at	*DNAH1*	45.2	15
	1553133_at	*C9orf72*	10.1	19
	1569305_a_at	*NA*	9.9	18
	219644_at	*CEP83*	7.9	7
	225919_s_at	*C9orf72*	6.2	17
	1552816_at	*NXNL2*	5.4	2

RELA+, *RELA* fusion positive; YAP1+, *YAP1* fusion positive.

### NanoString probes hybridisation performance and final selection of marker genes

NanoString probes were produced for the selected candidate genes (see supplementary material, Table S1) and hybridised to ependymoma samples. The RELA+ and YAP1+ marker probes hybridised to supratentorial tumours, while the PFA, PFA1, PFA2, and PFB marker probes hybridised to PF ependymomas. One probe for the *DRD1* gene exhibited uniform low hybridisation across all ependymoma samples, and a probe for *ANGTPL6* showed high expression in both *RELA* fusion‐positive and *RELA* fusion‐negative reference samples. Therefore, the probes for *DRD1* and *ANGTPL6* were excluded from subsequent clustering analyses.

In summary, the following marker genes were used for subsequent analysis in supratentorial tumours: *RELA*, *ELL3*, *FBP2*, *PCP4L1*, and *MYO3A* for detection of RELA+ tumours; and *MRAP*, *IGF1*, *CAPS*, and *WWC1* for detection of YAP1+ tumours. Likewise, the following marker genes were selected for analysis in PF tumours: *LAMA2*, *ALDH1L1*, *SLC6A13*, *IGSF1*, and *CXorf67* for PFA; and *NELL2*, *DNAH1*, *CEP83*, *C9orf72*, and *NXNL2* for PFB tumours.

### Clustering of tumours according to the expression levels of marker genes

#### Supratentorial ependymomas

**Figure 1:**
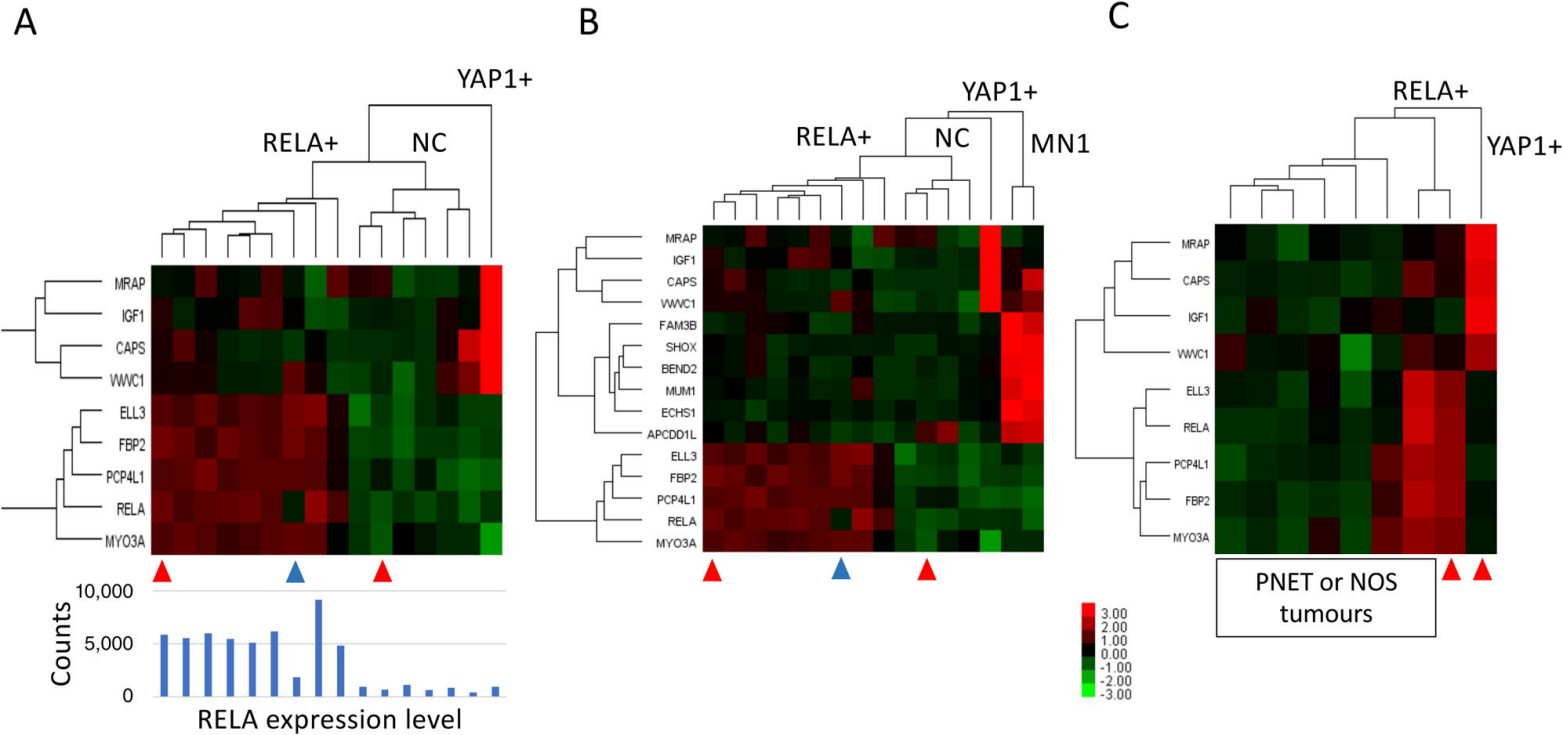
Clustering of supratentorial tumours according to expression levels of marker genes using the NanoString method. (A) Clustering of 16 ependymomas using nine signature genes reveals nine tumours with a RELA fusion‐positive signature, and one tumour with a YAP1 fusion‐positive signature. RELA expression levels are presented below the clusters. (B) Clustering of the same cohort of 16 tumours using 15 signature genes reveals two tumours with presence of the CNS HGNET‐MN1 signature. (C) Clustering of seven CNS‐PNET or CNS embryonal tumours NOS reveals one tumour with a RELA fusion‐positive signature. This tumour was diagnosed as CNS‐PNET prior to introduction of the WHO 2016 classification. Red arrowheads indicate the reference ependymoma samples. Blue arrowheads indicate tumours without *ZFTA‐RELA* fusion. Heatmap colours represent log2 gene expression differences. RELA+, *RELA* fusion‐positive signature; YAP1+, *YAP1* fusion‐positive signature; NC, not classified; MN1, CNS HGNET‐MN1 tumour.

Clustering analysis was performed using nine signature genes in 16 ependymomas. The results revealed two main clusters: cluster 1, which included nine tumours with high expression of RELA+ signature genes; and cluster 2, which contained six tumours without the expression of signature genes. Cluster 1 included the reference *RELA* fusion‐positive sample, and cluster 2 included the *RELA* fusion‐negative reference sample, as expected. One tumour showing expression of YAP1+ signature genes was separated from the remaining tumours. Tumours from the RELA+ cluster exhibited high *RELA* gene expression, with the exception of one sample that showed low *RELA* expression (Figure 1A).

Therefore, we designated nine samples from cluster 1 as RELA+, six samples from cluster 2 as not classified (NC), and one sample as YAP1+ ependymoma. Among the six NC samples, two tumours had been previously identified as CNS HGNET‐MN1 by molecular analysis [9]. To confirm their distinctive molecular identity, we repeated the clustering analysis with an additional six probes representing the CNS HGNET‐MN1 signature, which revealed clear separation of those two samples from the remaining tumours (Figure 1B). Therefore, NanoString analysis of histopathologically diagnosed ependymomas could molecularly classify 12 out of 16 tumours in our series, leaving four samples as NC ependymomas.

#### Supratentorial PNET or CNS embryonal tumours NOS

Ependymomas have previously been identified through gene methylation profiling among histologically diagnosed PNETs [10]. Therefore, we investigated whether NanoString‐based expression profiling was also helpful for the detection of ependymomas and their molecular subtypes. The RELA+ and YAP1+ signature probes were used to analyse seven histopathologically diagnosed PNET or CNS embryonal tumours NOS that were not previously characterised at the molecular level. Clustering analysis, including one RELA+ and one YAP1+ ependymoma for comparison, revealed one PNET sample displaying clear expression of RELA+ signature genes (Figure 1C).

#### PF ependymomas

**Figure 2:**
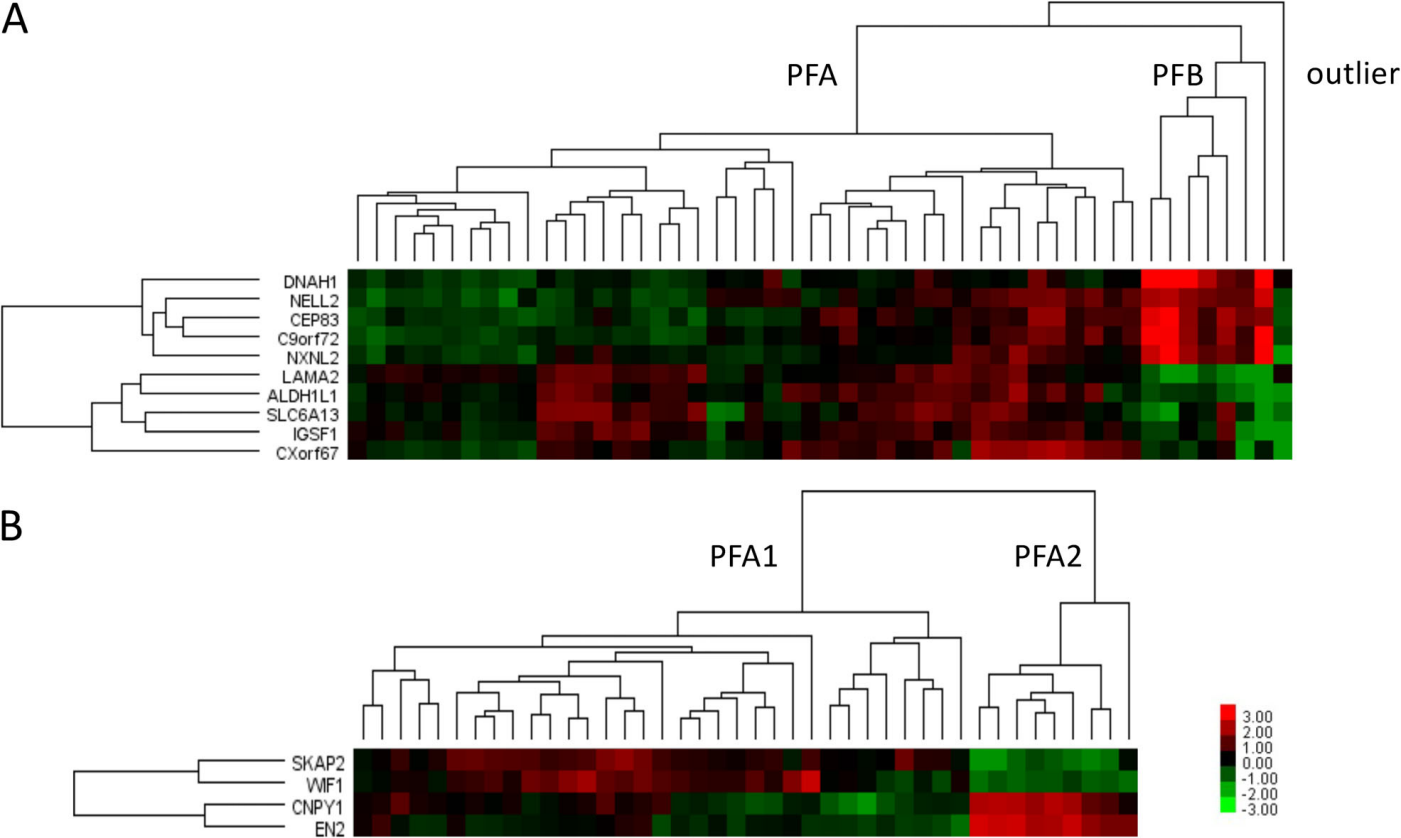
Clustering of PF ependymomas using the NanoString method. (A) Clustering of 50 ependymomas reveals 7 tumours with a PFB signature, 43 tumours with a PFA signature, and one outlier. (B) Clustering of 42 PFA ependymomas reveals 33 tumours with PFA1 signatures and 9 tumours with PFA2 signatures. Heatmap colours represent log2 gene expression differences.

Clustering was performed on 50 ependymomas using 10 signature genes. We identified two major clusters based on the expression of five PFA and five PFB genes (Figure 2A). Cluster PFB, which comprised 7 tumours, was clearly separated from the 42 PFA tumours. One sample was classified as an outlier, probably not an ependymoma. Additional analyses were performed only within the PFA cluster, using two PFA1 and two PFA2 signature genes. Two distinct clusters were identified: PFA1 with 33 samples and PFA2 with 9 samples (Figure 2B).

**Figure 3:**
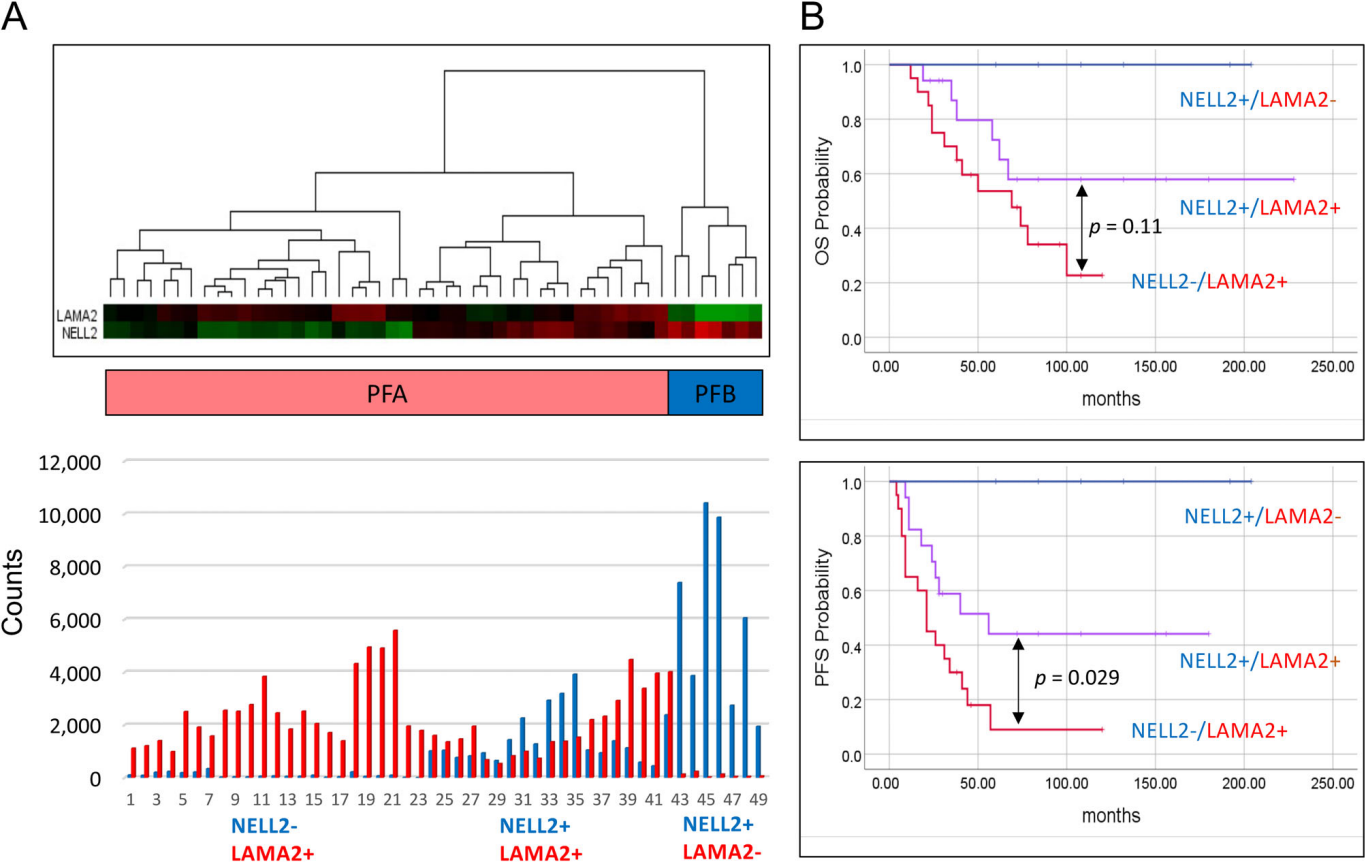
Ependymoma tumours subdivided according to *NELL2* and *LAMA2* expression status. (A) Clustering of 49 ependymomas reveals three clusters: NELL^+^/LAMA^−^, NELL+/LAMA+, and NELL−/LAMA+ tumours. Heatmap colours represent log2 gene expression differences. (B) Kaplan–Meier curves according to *NELL2* and *LAMA2* expression status. *P* values were calculated using the log‐rank test.

Subsequently, we clustered all samples using only two marker genes: *LAMA2* and *NELL2*. This revealed three main clusters. The first showed seven tumours with expression of *NELL2* but not *LAMA2* (NELL2+/LAMA2−), which overlapped exactly with tumours from the PFB cluster. The second cluster included 23 tumours showing expression of *LAMA2* but not *NELL2* (NELL2−/LAMA2+). The third cluster included 18 tumours that showed expression of both *NELL2* and *LAMA2* (NELL2+/LAMA2+). The second and third clusters overlapped with the PFA tumours (Figure 3A).

### Detection of *RELA* and *YAP1* fusion transcripts

NGS analysis was performed in all supratentorial tumours. In eight ependymomas and one PNET, which all showed the RELA+ NanoString signature, we detected the presence of the *ZFTA‐RELA* fusion transcript. In one ependymoma that exhibited the RELA+ signature, but low *RELA* expression at the RNA level (Figure 1A), we did not detect the *ZFTA‐RELA* fusion transcript. Among four ependymomas that were not classified by NanoString, and were analysed using the Archer FusionPlex Solid Tumour Panel, none exhibited the *ZFTA‐RELA* fusion, but one tumour showed the presence of the *ZFTA‐MAML2* fusion. Therefore, in our series, the presence of the NanoString RELA+ signature was significantly associated with the *ZFTA‐RELA* fusion (*p* = 0.005, Fisher's exact test). In the only sample showing the YAP1+ NanoString signature, we detected the *YAP1‐MAMLD1* fusion transcript. Table 2 presents the NGS results for individual patients.

**Table 2:** Characteristics of patients diagnosed with supratentorial tumours according to molecular group.

ID	Age (years)	Sex	NanoString diagnosis	NGS fusion	Original diagnosis/WHO stage	Relapse months/location	DOD (months)	ADF (months)	Primary treatment PPNG protocols	
									RT	Chemotherapy
1	12	M	RELA+	*ZFTA‐RELA*	EPN III	No	No	168	Local	EPN
2	12	F	RELA+	*ZFTA‐RELA*	EPN III	No	No	168	Local	EPN
3	5	M	RELA+	*ZFTA‐RELA*	EPN III	No	No	120	Local	EPN
4	3	M	RELA+	*ZFTA‐RELA*	EPN III	No	No	60	Local	EPN
5	6	M	RELA+	*ZFTA‐RELA*	EPN III	No	No	77	Local	EPN
6	2	F	RELA+	*ZFTA‐RELA*	EPN III	134/distant	No	168	No	For children <3 years old
7	11	F	RELA+	Not detected	EPN III	No	No	72	Local	EPN
8	12	M	RELA+	*ZFTA‐RELA*	EPN III	46/local	No	288	Local	No but EPN on relapse
9	14	M	RELA+	*ZFTA‐RELA*	EPN III	No	No	24	Local	EPN
10	1	M	RELA+	*ZFTA‐RELA*	PNET	22	42	–	No	MB/PNET
11	7	F	YAP1+	*YAP1‐MAMLD1*	EPN II	No	Yes	–	CSI	EPN
12	1	M	NC	*ZFTA‐MAML2*	EPN II	No	No	156	No	For children <3 years old
13	17	F	NC	Not detected	EPN III	No	No	168	Local	EPN
14	16	F	NC	Not detected	EPN II	No	No	216	Local	EPN
15	2	M	NC	Not detected	EPN III	No	No	96	No	For children <3 years old

ADF, alive disease free; CSI, cerebrospinal irradiation; DOD, died of disease; EPN, ependymoma; MB, medulloblastoma; NC, not classified; PPNG, the Polish Paediatric Neurooncology Group; RELA+, *RELA* fusion positive; RT, radiotherapy; YAP1+, *YAP1* fusion positive.

### Histopathological characteristics

**Figure 4:**
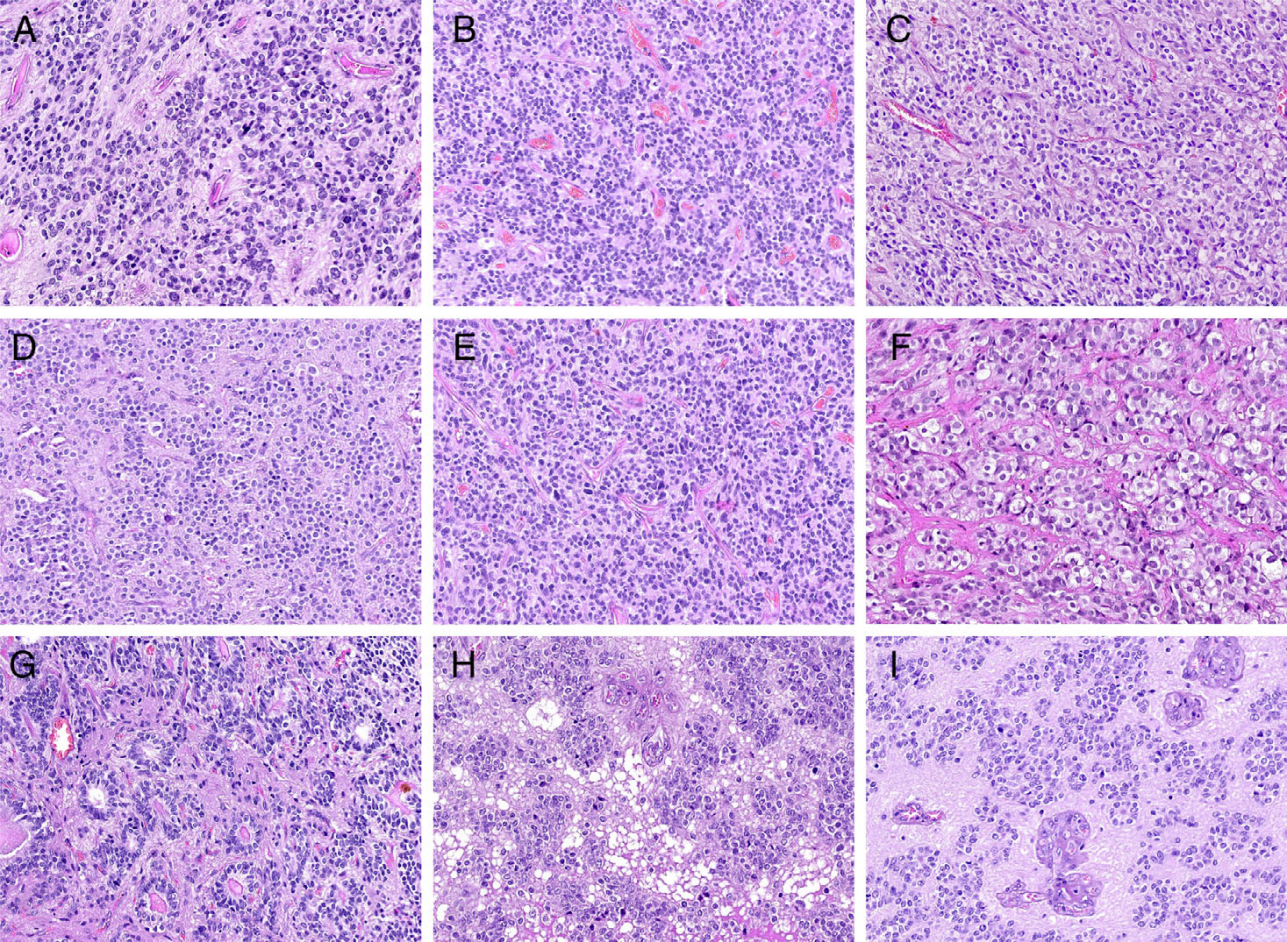
Representative histopathology of *RELA* fusion‐positive ependymomas. (A) A tumour of moderate cellularity accompanied by perivascular pseudorosettes. (B) Abundant small blood vessels surrounded by indistinct perivascular anucleate zones. (C) High density of neoplastic cells with clear‐cell morphology. (D, E) Oligodendrocyte‐like pattern with honeycomb appearance and prominent network of thin‐walled branching capillaries. (F) Sheets of closely packed monomorphic cells with clear, empty, or slightly eosinophilic cytoplasm. (G) True ependymal rosettes comprising tumour cells surrounding a central lumen. (H) Area with microcystic changes and a population of uniform cells with small clear perinuclear halos. (I) Microvascular proliferation associated with wide anucleate perivascular zones. Haematoxylin and eosin staining. Original objective magnifications: A, F: ×30; B–E, G–I: ×20.

Histologically, the *RELA* fusion‐positive ependymomas appeared as solid or solid/focal cystic lesions. We commonly found perivascular pseudorosettes, which typically comprised neoplastic cells radially orientated around the blood vessels, with more‐or‐less distinctive perivascular anucleate zones (Figure 4A,B). In more compact areas, pseudorosettes were sparse and indistinct. The tumours frequently exhibited clear‐cell morphology, which was the dominant cytopathological pattern or was at least focally visible (Figure 4C,D). Some parts of the tumours exhibited a honeycomb architecture, accompanied by a network of thin‐walled branching capillaries, resembling classic oligodendrogliomas (Figure 4E). We also observed sheets of tightly packed monomorphic cells with a centrally located round‐to‐oval nuclei, surrounded by empty cytoplasm (Figure 4F). Ependymal tubes were only occasionally seen, and true ependymal rosettes were found in only one case (Figure 4G). We observed focal perivascular hyalinisation and microcystic changes (Figure 4H). Microcalcifications were frequently seen. All cases exhibited mitotic figures, microvascular proliferation (Figure 4I), and necrosis, corresponding with a histological diagnosis of WHO grade III anaplastic ependymomas.

**Figure 5:**
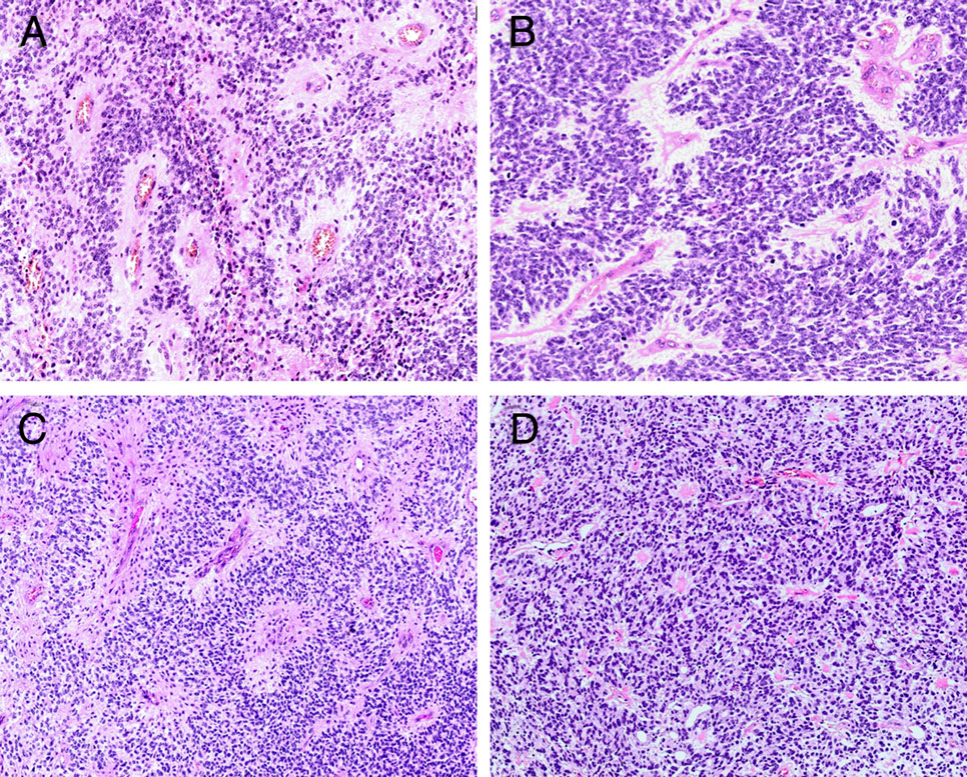
Histopathology of PF ependymomas. (A, B) PFA tumours with pronounced perivascular rosettes. (C) PFB tumour with prominent perivascular pseudorosettes. (D) PFB tumour with high vascularity and small anucleate perinuclear zones. Haematoxylin and eosin staining. Original objective magnifications: A, B: ×20; C, D: ×15.

All PF ependymomas, classified as PFA and PFB tumours WHO grade II and III, exhibited morphological heterogeneity; however, we found no significant morphological differences between these groups. Distinct perivascular pseudorosettes were a specific histopathological feature of both PFA (Figure 5A,B) and PFB (Figure 5C,D) ependymomas. We occasionally observed delicate branched capillaries and clear‐cell appearance in the neoplastic tissue. Nodules of high cell density within regions of low cell density background were sometimes seen. Anaplastic lesions exhibited dense cellularity, microvascular proliferation, necrosis, and mitotic figures.

### Clinical characteristics of patients with supratentorial tumours

We detected nine RELA+, one YAP1+, and four not molecularly classified ependymomas. Table 2 presents the patients' clinical characteristics. None of the patients presented with metastases, except the YAP1+ patient, who exhibited lesions in the spinal cord that were suspicious for disease metastases. All patients underwent gross total resection of tumours and were treated according to the Polish Paediatric Neurooncology Group (PPNG) protocols. All patients responded well to the chemotherapy and/or radiotherapy they received. Seven RELA+ ependymoma patients received both radiotherapy and chemotherapy during the primary treatment, and none have relapsed or died. The other two RELA+ patients relapsed, but are still alive at ≥10 years after diagnosis. The only RELA+ infant patient relapsed and died. He had been originally diagnosed with PNET and was treated according to the medulloblastoma/PNET protocol.

### Clinical characteristics of patients with PF ependymomas

**Figure 6:**
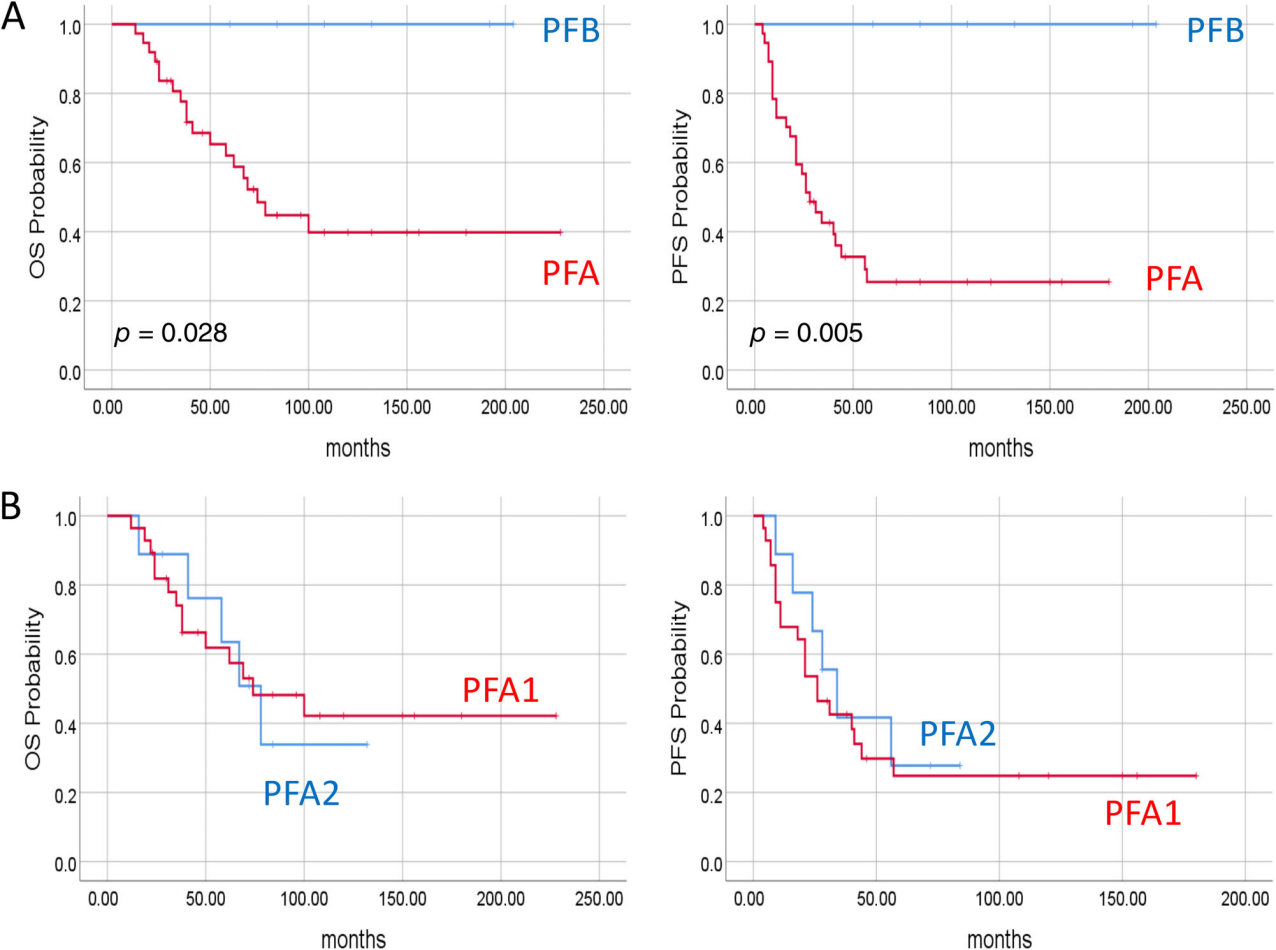
Survival of patients with PF ependymomas according to molecular groups. (A) Kaplan–Meier curves for PFA and PFB ependymomas. (B) Kaplan–Meier curves for PFA1 and PFA2 ependymomas. *P* values were calculated using the log‐rank test.

Clustering analysis identified 42 PFA and 7 PFB patients. Patients with PFB were significantly older than patients with PFA tumours (mean ages of 8.2 and 3.9 years, respectively; *p* = 0.001). The groups did not differ significantly in terms of gender, WHO classification of the tumour, or frequency of metastases. We did observe a significant difference in survival rate. The seven patients from the PFB group did not relapse or die of disease, even though four of them were treated with only radiotherapy. Compared to patients with PFA ependymomas, patients with PFB tumours showed significantly higher OS (*p* = 0.025) and PFS (*p* = 0.005) (Figure 6A).

Clustering analysis within the PFA group identified 33 patients with PFA1 and 9 patients with PFA2 tumours. These groups did not differ significantly in terms of age, WHO classification, or survival rate (*p* = 0.88 for OS and *p* = 0.62 for PFS) (Figure 6B). All patients with PFA2 tumours were males, which was the only significantly different feature in this cohort (*p* = 0.02).

Additional survival analyses revealed significant differences based on *NELL2* and *LAMA2* expression status. The 5‐year survival rate was best among NELL2+/LAMA2− patients (100% OS and PFS), medium among NELL2+/LAMA2+ patients (72% OS and 53% PFS), and worst for NELL2−/LAMA2+ patients (44% OS and 9% PFS) (Figure 3B). The NELL2−/LAMA2+ patients were significantly younger than the NELL2+/LAMA2+ patients (mean age of 2.04 versus 5.6 years, *p* = 0.001).

## Discussion

In this study, we applied a novel approach for the diagnosis of four molecular groups of ependymoma, using the NanoString nCounter Analysis System and multi‐gene group‐specific signatures. This analysis is both reliable and cost effective due to the use of a limited but specific number of probes (4–5 for each group) that detect highly expressed marker genes. As mentioned in our previous paper [9], it is important that reference samples from molecularly diagnosed tumours are included in the analysis of new cases. The complete diagnostic process – including RNA extraction, hybridisation, and RCC file analysis using the freely available nSolver software – can be accomplished within 3 days.

Examination of supratentorial ependymomas, to identify RELA+ and YAP+ tumours, was based on the simultaneous analysis of nine marker genes. Clustering analysis revealed nine RELA+, one YAP1+, and six unclassified tumours. Among the unclassified tumours, two samples were identified as CNS HGNET‐MN1 tumours through the use of six additional tumour‐specific marker genes. This result confirms previous findings that some histologically diagnosed ependymomas belong to this newly described molecular entity [10]. Overall, four ependymomas were not classified in our analysis. We identified only one YAP1+ tumour, confirmed by the presence of the *YAP1‐MAMLD1* fusion, in an infant girl, indicating that this molecular type of ependymoma is relatively rare. Indeed, in a series of 29 histopathologically verified ependymomas, Fukuoka *et al* [13] also identified only one *YAP1* fusion‐positive tumour. The remaining eight tumours lacking a *RELA* or *YAP1* fusion presented as a heterogenous cohort, supporting our findings that paediatric ependymomas include tumours that can be classified as *RELA/YAP1* fusion‐negative. Such tumours may show other genetic abnormalities; indeed, we have identified a *ZFTA‐MAML2* fusion in an infant boy who is a long‐term survivor. Very recently, a similar fusion was described in an anaplastic ependymoma from a 23‐year‐old man, who has been alive for 30 months without any evidence of disease [14], as well as in a 2‐year‐old boy with unknown disease outcome [15].

In our present study, the majority of supratentorial ependymomas (9 out of 14) belonged to the RELA+ category, confirming previous reports of the high frequency of this molecular type [1, 2, 13]. In all but one analysed case, the NanoString signature correlated significantly with the presence of a *ZFTA‐RELA* fusion. Our series showed one discrepancy where the RELA+ signature occurred without a *RELA* fusion. Similarly, Tamai *et al* [14] and Zschernack *et al* [15] have described cases in which tumours with an ‘ependymoma, *RELA* fusion’ positive methylation signature exhibited alternative fusions rather than the *ZFTA‐RELA* fusion. The authors suggested that the methylation‐based classification seems insufficient to distinguish *RELA/YAP1* fusion‐negative cases. On the other hand, our expression‐based RELA+ signature could distinguish *RELA* fusion‐positive cases from *RELA* fusion‐negative cases exclusively based on the expression level of the *RELA* gene. Nevertheless, such peculiar and rare cases require further investigation in terms of both the biological mechanism and clinical impact on patients.

From a clinical perspective, it is crucial to identify *RELA* fusion‐positive tumours, as initial data have suggested a poor prognosis for patients [2]. However, a recent analysis of *RELA* fusion‐positive patients treated following HIT2000‐E protocols demonstrated good OS for patients without *CDKN2A* deletion and retained p16 protein. Worse survival was observed among patients with homozygous *CDKN2A* deletion (16.7% of patients) [16]. In our present study, there was a 100% survival rate among the seven RELA+ patients who were uniformly treated, and older than 3 years of age. It is very likely that the majority or all of these patients do not carry the uncommon homozygous *CDKN2A* deletion. Our results highlight that a large proportion of *RELA* fusion‐positive patients may have a good prognosis. The impact of treatment should also be considered, as our patients received both radiotherapy and chemotherapy.

The only *YAP1* fusion‐positive tumour was detected in an infant girl. She displayed severe toxicity during treatment and later died. The four *RELA/YAP1* fusion‐negative ependymoma patients varied in terms of age (1–17 years old) and treatment. Two infants did not receive radiotherapy, but all four patients are long‐term survivors after at least 8 years of observation, including a *ZFTA‐MAML2* fusion‐positive patient. Clearly, our findings demonstrate the existence of *RELA/YAP1* fusion‐negative patients, in agreement with previous descriptions of similar cohorts of patients [14, 15].

We identified two main clusters of infratentorial ependymomas. The smaller cluster (14.2% of tumours) showed high expression of five genes belonging to the PFB signature and NELL2+/LAMA2− expression pattern. This type of tumour was less frequent in our paediatric series, in contrast to previously published data including adult patients [2, 3, 17]. The PFB ependymoma group identified in our present study comprised older children with good prognosis, confirming previous findings. Tumours in the bigger PFA cluster were clearly separated from the PFB cluster and showed heterogeneous patterns. The molecular heterogeneity of PFA tumours was recently described by Pajtler *et al* [7], who noted two major subgroups (PFA1 and PFA2) and nine minor subgroups. We subdivided PFA tumours into PFA1 and PFA2 subgroups using four marker genes. This classification did not show any significance in terms of survival rate, similar to the findings of Pajtler *et al* [7].

Witt *et al* [3] described the immunohistological categorisation of PF ependymomas using NELL2 and LAMA2 antibodies. We performed a similar analysis, with assessment of the RNA expression levels of these genes. We identified three major clusters, with the NELL2+/LAMA2− pattern overlapping with PFB ependymomas, and the remaining two clusters overlapping with PFA ependymomas. NELL2−/LAMA2+ patients were mainly infants (74%) and showed a particularly poor survival rate, while only 15% of NELL2+/LAMA2+ patients were infants and these patients showed a medium survival rate. PFS differed significantly between these two groups. It is likely that the different age distribution may have impacted the modes of treatment, thereby influencing patient survival. Nevertheless, our different methodological approach yielded survival data analogous to those presented by Witt *et al* [3], supporting the clinical heterogeneity among PFA ependymomas and the need for additional prognostic markers.

The recent cIMPACT‐NOW (the Consortium to Inform Molecular and Practical Approaches to CNS Tumor Taxonomy) update 6 [18] highlights the use of immunohistochemistry for H2K27me3 as a marker for PF ependymomas [19]. PFA ependymomas are diagnosed by loss of nuclear H3 K27me3 expression, in contrast to PFB tumours. This method is straightforward and correlates with the DNA methylation profiles of tumours. Nevertheless, formal correlation between K27me3 expression and methods based on gene expression profiling, including NanoString classification, requires further investigation.

Notably, the NanoString‐based approach can be used to identify PFA and PFB ependymomas, and may also provide further opportunity for the detection of heterogeneity among PFA tumours. The NanoString method is based on an open system, and additional marker genes can be added to the custom set to establish biological or prognostic subgroups in future research.

In summary, we confirm here that the NanoString approach is a useful tool for the diagnosis of all four main molecular groups of ependymomas. This single methodology can detect molecular types characterised by either the presence of oncogenic fusions or the involvement of epigenetic mechanisms and lack of recurrent somatic mutations. From a clinical perspective, further research is required to assess the impact of *ZFTA‐RELA* and other fusions on patient survival, as well as the clinical and biological heterogeneity among *RELA/YAP1* fusion‐negative and PFA ependymomas.

## Author contributions statement

MŁ designed the study, performed data analysis and interpretation, and wrote the manuscript. EM performed the histopathological examination of tumours, and was a major contributor to the writing of the manuscript. BW performed the bioinformatics analyses of microarray data. ASo, MN, ASz, AK‐W, MK, ME and JT carried out the experiments and data analysis. AK contributed to implementation of the project based on NanoString technology. MT, MP‐P and BD‐B collected and interpreted patients' clinical data. All authors were involved in writing the paper and approved the submitted and published versions.

## Supporting information


**Table S1.** Target regions of the marker genes
