# Supplementary material for: Transcriptional profiling of paediatric ependymomas identifies prognostically significant groups
Source: J Pathol Clin Res. 2021 Jul 27;7(6):565–76. doi: 10.1002/cjp2.236 (PMC8503892; doi:10.1002/cjp2.236)
Supplement: Supplementary file 1 — Table S1. Target regions of the marker genes [file CJP2-7-565-s001.pdf]

Transcriptional profiling of paediatric ependymomas identifies prognostically significant groups

M Łastowska, E Matyja *et al*. *J Pathol Clin Res* DOI: 10.1002/cjp2.236

Table S1. The target regions of the marker genes in the molecular groups of ependymoma

The probes used in NanoString clustering analyses were designed to target the following regions of the marker genes:

Group	Gene	Accession	Target sequence
RELA+	*RELA*	NM_021975.4	TCCGCGGGCAGCATCCCAGGCGAGAGGAGCACAGATACCACCAAGACCCACCCCACCATCAAGATCAATGGCTACACAGGACCAGGGACAGTGCGCATCT
	ELL3	NM_025165.2	CACTGCCTTCCTCTGCCAGCCGGAAACGTCTGGACAAGAAACGTTCAGTGCCTGTAGCCACTGTAGAACTGGAAGAAAAGAGGTTCAGAACTCTGCCTTT
	FBP2	NM_003837.2	AGATGAGGTGAAGAAACTGGATGTGCTATCCAATTCCCTGGTGATCAACATGGTCCAATCCTCCTATAGTACCTGCGTCCTGGTCTCAGAAGAGAATAAG
	PCP4L1	NM_001102566.2	GTAGATTAGTTCCAAGGAAGGGAGACTGGAATGCTGGTGTCAAGGAAAAGCCTCCCTCATCATCTAGTCTAAGACCATAACGGGCAGAAGCATAAGAGGT
	MYO3A	NM_017433.5	TGGCCAACAGAACACTTGCTAGCGGTTGAATCTTAGAGAAAAAAGCCCGGGAGGGGTGGGGAGAATTTCGAAGATGTATTTCATCTCAAGCTTGCTCTTT
YAP1+	*CAPS*	NM_080590.2	TGGAGGCCATCCTGGAAGGAAGTTTAGACCTGCCCAGGTGTGGAGCGAGGGGCACAGGGGCATCCTAACCTCAGAAACTGAAATAAAGCCTTTGAAAAAA
	WWC1	NM_015238.2	AGCCACCTTTTGTTCGAAACTCCCTGGAGCGACGCAGCGTCCGGATGAAGCGGCCTTCCTCGGTCAAGTCGCTGCGCTCCGAGCGTCTGATCCGTACCTC
	IGF1	NM_000618.3	CGTGGATGAGTGCTGCTTCCGGAGCTGTGATCTAAGGAGGCTGGAGATGTATTGCGCACCCCTCAAGCCTGCCAAGTCAGCTCGCTCTGTCCGTGCCCAG
	MRAP	NM_206898.1	CATACTACAGCTATGAATACTACCTGGACTATCTGGACCTCATTCCCGTGGACGAGAAGAAGCTGAAAGCCCACAAACATTCCATCGTGATCGCATTCTG
PFA	*LAMA2*	NM_000426.3	GGCAAACTCAAGTATGCAATCTATTTCGAGGCTCGGGAAGAAACAGGTTTCTCTACATATAATCCTCAAGTGATCATTCGAGGTGGGACACCTACTCATG
	ALDH1L1	NM_012190.2	AGAAGGATGGAGTGCCGGTATTCAAGTACTCCCGGTGGCGTGCAAAAGGACAGGCTTTGCCTGATGTGGTGGCAAAATACCAGGCTTTGGGGGCCGAGCT
	SLC6A13	NM_016615.4	TAGCAGGGTCTCAGGCACAACCAGTAATGGAGAGACAAAACCAGTGTATCCAGTCATGGAAAAGAAGGAGGAAGATGGCACCCTGGAGCGGGGGCACTGG
	IGSF1	NM_001170961.1	TCTCGGATATCAAGCAAGTTCCTGCTGCTGAAGGATAAGACACAGATGACCTGGATCCGCCCTTCCCACAAGACCTTCCAAGTTTCATTCCTTATAGGTG

	CXorf67	XR_113306.1	GAGTTTTTGGGCCTGAGATCTATCTCCACTCCTAGCCCTGAAAGCCTTAGGTATGCTTTGATGCCTGAGTTTTATGCTCTGAGCCCTGTCCCTCCAGAAG
PFA1	*SKAP2*	NM_003930.3	TCTTAGCAAGGAATACAATAGATATGGCTGGTGGGTAGGAGAAATGAAGGGAGCCATTGGCTTGGTGCCTAAAGCCTACATAATGGAGATGTATGATATT
	WIF1	NM_007191.2	ACGATGTATGAATGGTGGACTTTGTGTGACTCCTGGTTTCTGCATCTGCCCACCTGGATTCTATGGAGTGAACTGTGACAAAGCAAACTGCTCAACCACC
PFA2	*EN2*	NM_001427.3	CACACACCAGGCGTGTTTGAGTCCACAGTTCTGAAACATGTGGCTACCTTGTCTTTCAAAAGAACTCAGAATCCTCCAGGATCTAGAAGAAGGAAGAAAG
	CNPY1	NM_001103176.1	CTGTGTGAAACTTCTGCTAATCATACTGAGCTCTAGGAGTGCCGTTCTGCTGGTCGTAGAGAGGAGTAAAGTCACACCCAAAGGTCAATGTCTGCATTTT
PFB	*NELL2*	NM_006159.1	CAGATTGACGTCTTAACAGAGTTAGAACTTGGGGAGTCCACGACCGGAGTGCGTCAGGTCCCGGGGCTGCATAATGGGACGAAAGCCTTTCTCTTTCAAG
	DNAH1	NM_015512.4	TTGGCGAGACATCCTAGCACTCTCGAACCTGCCAACCTTTTCCTCCTTCTCTTCCGACTTCGTGAAGCACCTCTCAGAATTCCGGGTCATCTTCGACAGC
	NXNL2	NM_001161625.1	GGGTGCCGCGCTGTCGCCCAGGTATCTGGGGTCTCTGGTGTCTGAGTGTCTCATTGTCGGCGCGAACACAATTGCTCCAGCCACAGGCGAGGCCTGGCCA
	C9orf72	NM_145005.6	GAGTAAGGCACATTTGGGCTCCAAAGACAGAACAGGTACTTCTCAGTGATGGAGAAATAACTTTTCTTGCCAACCACACTCTAAATGGAGAAATCCTTCG
	CEP83	NM_016122.2	TCAGAGATTGCAAGACTGGAGGAAGATAAAGAAGAACTACGTAACCAGCTGCTTAATGTTGATCTCACAAAAGACAGCAAACGAGTGGAACAACTTGCTC

RELA+ - *RELA* fusion positive, YAP1+ - *YAP1* fusion positive
